# A Case of Myocardial Infarction in a Patient With Spontaneous Spinal Subdural Hematoma

**DOI:** 10.7759/cureus.37929

**Published:** 2023-04-21

**Authors:** Zhongying Liu-An, Vladimir Joseph, Stacey Damito

**Affiliations:** 1 Medicine, Hackensack University Medical Center, Hackensack, USA; 2 Cardiology, Hackensack University Medical Center, Hackensack, USA

**Keywords:** oxygen supply-demand mismatch, type 2 mi, type 1 mi, ssdh, spontaneous spinal subdural hematoma

## Abstract

Spontaneous spinal subdural hematoma (SSDH) is an extremely rare clinical condition and a neurologic emergency that is most commonly associated with anticoagulation therapy and coagulopathy. We present a case of myocardial infarction (MI) with an unusually high troponin level in the setting of SSDH. This case underscores the challenges and importance of properly differentiating type 1 MI from type 2 MI as the management is different. It also presents challenges in managing MI in the setting of recent bleeding in the context of desired anticoagulation and antiplatelet therapy.

## Introduction

Spontaneous spinal subdural hematoma (SSDH) is a rare clinical condition accounting for approximately only 4.1% of all spinal hematomas [1]. It occurs within the subdural space and most frequently in the thoracic spine. Patients usually present with sudden onset back pain that may radiate down the lower extremities along with motor, sensory, and automatic abnormalities [2]. SSDH is a neurological emergency that can lead to rapidly progressive neurological deficits due to spinal cord compression. The etiology remains unclear as the subdural space lacks bridging veins as a source of bleeding. There are three major risk factors of SSDH including spontaneous, trauma, and iatrogenic causes [3] with spontaneous being extremely rare [4]. Magnetic resonance imaging (MRI) is the diagnostic choice. The prompt diagnosis followed by surgical decompression, percutaneous drainage, or conservative management can lead to potentially full clinical recovery [5].

The Fourth Universal Definition of Myocardial Infarction (UDMI) published in 2018 defined myocardial injury as an elevation of cardiac troponin above the 99th percentile upper reference limit (URL) of 0.04 ng/mL [6]. Myocardial injury can be acute or chronic. Acute myocardial injury presents with rising or falling troponin levels in a series of tests, while chronic myocardial injury has stable troponin levels over time. Among patients with an acute myocardial injury who have evidence of myocardial ischemia including acute coronary symptoms, new ECG findings, and imaging abnormalities, the diagnosis of acute myocardial infarction (MI) is established [7]. There are five types of MI based on the Fourth UDMI [6]. Type 1 is due to the acute coronary atherothrombotic event with plaque rupture. Most patients with ST-segment elevation myocardial infarction (STEMI) and many with non-ST-segment elevation myocardial infarction (NSTEMI) fit into this category. Type 2 is due to acute myocardial ischemia from either increased oxygen demand or decreased oxygen supply. Type 3 happens when sudden cardiac death occurs before the troponin level is measured or before the troponin elevation is detected. Type 4 is associated with percutaneous coronary intervention (PCI) restenosis, and type 5 is related to coronary artery bypass graft (CABG).

Treatment for type 1 MI has been well established including aspirin, sublingual nitroglycerin, oxygen supplementation, and analgesics with possible thrombolytic therapy or primary percutaneous transluminal coronary angioplasty [8]. Type 2 MI treatment focuses on treating underlying causes of oxygen supply-demand mismatch.

## Case presentation

**Figure 1:**
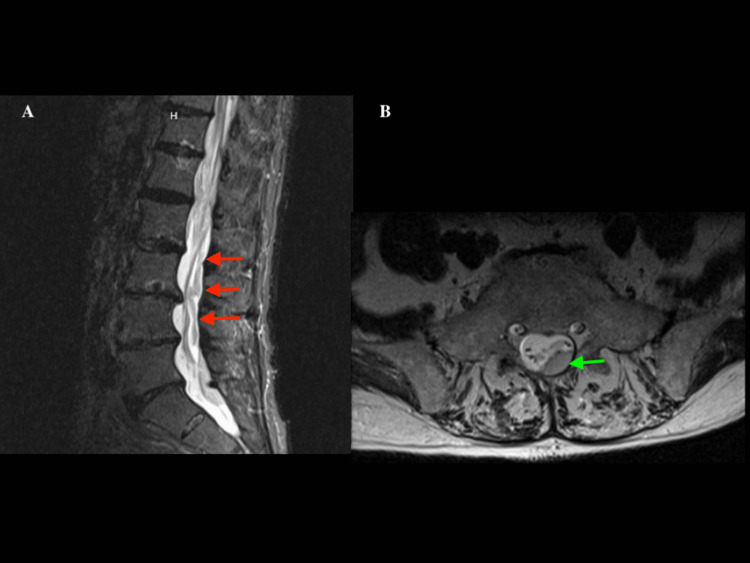
MRI of the lumbar spine without contrast. A: Sagittal view shows extensive subdural hematoma in the lumbar spine (red arrows). B: Axial view shows subdural hematoma in L5 level (green arrow). MRI: magnetic resonance imaging.

An 89-year-old male with a past medical history of coronary artery disease requiring CABG in 1993 and PCI in 2012, chronic atrial fibrillation (AF) on dabigatran, right bundle branch block (RBBB), hypertension, and hyperlipidemia presented with sudden onset back pain radiating down his right leg, bilateral lower extremity weakness and numbness, and acute-onset urinary retention. On presentation, he was afebrile and hypertensive to 241/128 mmHg. Physical exam revealed irregularly irregular rhythm, no tenderness on palpation of the spine, and diminished sensation and 0/5 strength of the right lower extremity. The complete blood count was within normal limits. Initial troponin was 0.03 ng/mL (reference: 0-0.04 ng/mL). Electrocardiogram (ECG) at presentation showed AF without rapid ventricular response (RVR), RBBB, and no ST-segment changes. MRI of the thoracic and lumbar spine without contrast showed a large SSDH extending from the cervical spine down to sacral levels, with severe spinal cord and cauda equina compression (Figures 1A, 1B). Dabigatran was held. The patient underwent an emergent T8-10 bilateral laminectomy and surgical SSDH evacuation.

**Figure 2:**
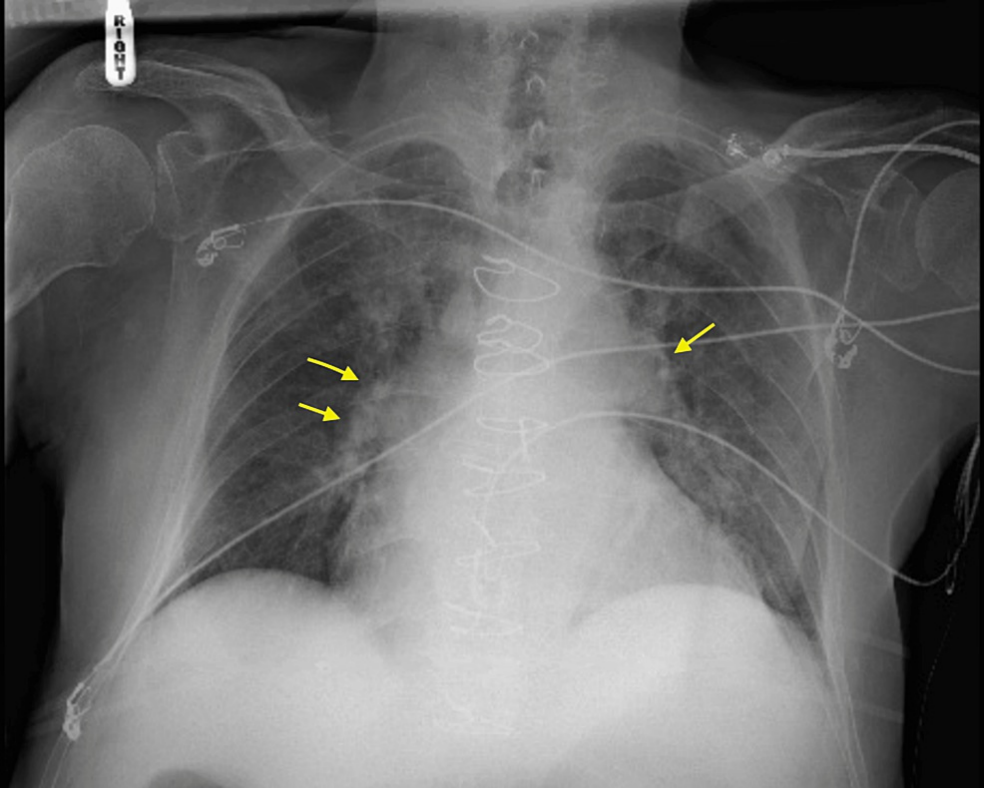
Initial chest X-ray on POD 1. Initial chest X-ray showed pulmonary congestion (yellow arrows). POD: postoperative day.

**Figure 3:**
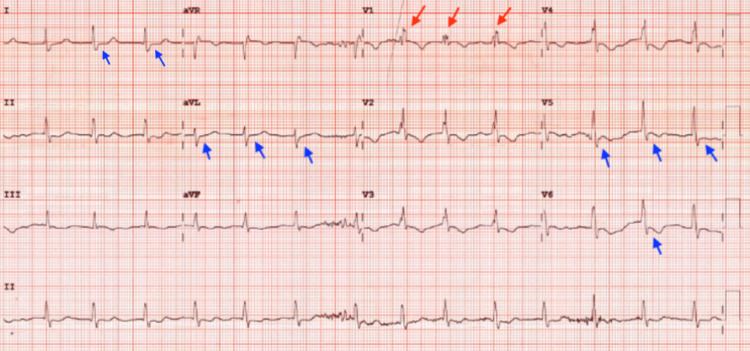
ECG on POD 1. Atrial fibrillation and right bundle branch block in lead V1 (red arrows), and ST-segment depression in lateral leads I, aVL, V5, and V6 (blue arrows). POD: postoperative day.

On postoperative day (POD) 1, the patient developed dyspnea and acute hypoxic respiratory failure with an arterial oxygen pressure of 44 mmHg on the arterial blood gas, for which he was placed on a high-flow nasal cannula at 50 L/50% fraction of inspired oxygen (FiO_2_). He denied having any chest pain. Systolic blood pressure was persistently over 180 mmHg despite treatment with intravenous labetalol. Morning labs were significant for troponinemia 3.25 ng/mL, lactic acidosis 4.0 mmol/L (reference: < 2 mmol/L), leukocytosis 15.3 × 10^9^/L (reference: 4.5-11.0 × 10^9^/L), and anemia 11.5 g/dl (reference: 13.8-17.2 g/dl in males). Sputum culture was collected. Chest X-ray showed pulmonary vascular congestion (Figure 2). Repeat ECG showed AF without RVR, RBBB, and ST-segment depression in lateral leads (Figure 3). Transthoracic echocardiogram (TTE) showed normal left ventricular ejection fraction (LVEF) at 55%-60% without regional wall motion abnormalities. Bilateral lower extremity venous Doppler exams were negative for deep vein thrombosis. Due to persistent hypertension, nicardipine infusion was started. 

**Figure 4:**
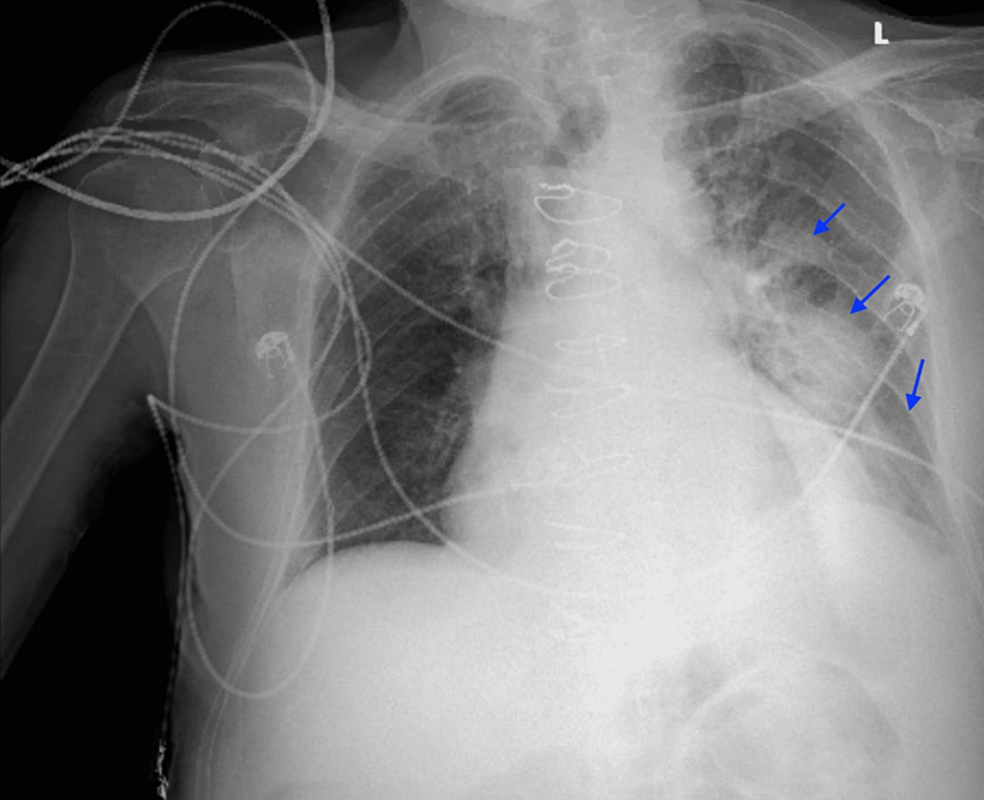
Repeat chest X-ray on POD 1. Repeat chest X-ray a few hours later on POD 1 showed increasing dense left perihilar and basilar consolidation, increasing patchy left pulmonary infiltrates, and small left pleural effusion that may reflect pneumonia and/or asymmetric edema (blue arrows). POD: postoperative day.

A few hours later, his arterial oxygen saturation decreased to 78%, but he was otherwise hemodynamically stable. Serial troponin trended up to 63.46 ng/mL. B-type natriuretic peptide (BNP) was 467 pg/mL (ref: <100 pg/mL). Repeat chest X-ray showed dense left perihilar and basilar consolidation, increasing patchy left pulmonary infiltrates, and a small left pleural effusion, which may have reflected developing pneumonia (Figure 4). Procalcitonin was 0.85 ng/mL (ref: <0.1 ng/mL). The patient was started on a five-day empiric course of ceftriaxone and doxycycline, and interventional cardiology was consulted for NSTEMI. The patient was ultimately determined to be a poor candidate for catheterization intervention due to recent SSDH and his age. A decision was made to continue with medical management using aspirin 325 mg daily, while anticoagulation was held.

On POD 2, troponin trended down to 39 ng/mL. Nicardipine infusion was discontinued as blood pressure normalized, and he was optimized on guideline-directed medical therapy (GDMT). Over the next few days, troponin levels continued to decrease. Sputum culture was positive for *Paenibacillus* species and *Corynebacterium amycolatum*. The patient completed five days of ceftriaxone and doxycycline. He was eventually weaned off supplemental oxygen and was subsequently discharged to subacute rehabilitation in a stable condition.

## Discussion

SSDH is a rare neurologic emergency consisting of only 4.1% of all intraspinal hematomas [9]. It requires early diagnosis, discontinuation of anticoagulation, and urgent surgical decompression. Among the three main causes of SSDH including spontaneous, trauma, and iatrogenic injury, spontaneous SSDH is the rarest. Studies have shown that SSDH is most commonly associated with anticoagulation therapy and coagulopathy [3,10]. In our case, long-term treatment with dabigatran for chronic AF had probably posed an increasing risk of SSDH to the patient. 

Chest pain is the most common presenting symptom in type 1 MI and is more frequently associated with type 1 MI than type 2 MI [11]. Although our patient did not have chest pain, he developed dyspnea, ST-segment depression in lateral leads on ECG, and a troponin elevation to 63.46 ng/mL, significantly above the 99th percentile of the upper reference limit. Along with his extensive cardiac history, he was at great risk of type 1 MI. A mainstay of treatment includes antithrombotic therapy and/or revascularization [12]. While type 1 MI remained our top diagnosis, this patient’s normal LVEF and absent regional wall motion abnormalities on TTE prompted us to further explore other underlying causes that might have contributed to his unusual troponin elevation.

Type 2 MI is caused by decreased oxygen supply or increased oxygen demand. The most common cause is operative stress, followed by sepsis, arrhythmia, heart failure, and anemia [13]. It is possible that operative stress played an important role in causing type 2 MI in this patient after major spinal surgery. In addition, his acute hypoxic respiratory failure, dyspnea, and preserved cardiac function on POD 1 are more prevalent in type 2 MI [11]. He may have also developed sepsis secondary to pneumonia. It is noted that a retrospective study found troponin elevation in a significant portion of patients with septic shock [14]. Although pulmonary embolism was less likely the cause of his troponin elevation due to the lack of right ventricular strain noted on TTE and negative lower extremity venous Doppler tests, it can be a major trigger of type 2 MI [15]. Factors that can cause oxygen supply-demand imbalance can also contribute. In his case, hypertensive urgency, AF, and anemia, all could have been contributory. 

The key to type 2 MI management is to treat reversible causes of oxygen supply-demand mismatch. Interestingly, about two-thirds of patients with type 2 MI have coronary artery disease and one-third have left ventricular systolic dysfunction [16]. As GDMT is associated with significantly reduced mortality in patients with coronary artery disease and heart failure with reduced ejection fraction [17], initiation of GDMT in addition to treating the underlying cause of oxygen supply-demand mismatch can provide the potential to improve clinical outcomes in patients with type 2 MI. While we could not further narrow down the cause, appropriate treatment with antibiotics, GDMTs, oxygen supplementation, and intravenous fluid resuscitation led to the patient’s significant clinical improvement.

This case also presented challenges in managing MI in the setting of recent bleeding. Revascularization with PCI would require dual antiplatelet therapy [18] which might be contraindicated in this patient given his recent history of spontaneous SSDH and laminectomy. Since the risk of hemorrhage outweighed the risk of ischemia, a decision was made to continue with conservative medical management with aspirin 325 mg and GDMT.

## Conclusions

There is a considerable overlap of clinical features and risk factors between type 1 and type 2 MI. The distinction between these two types is challenging when clinical symptoms of ischemia cannot be reliably detected. This case underscores the importance of properly delineating type 1 MI from type 2 MI as the management is different. Type 1 MI treatment focuses on antithrombotic therapy and/or revascularization, while type 2 MI targets treating the underlying causes of oxygen supply-demand imbalance. An elevation of troponin indicates the presence of myocardial injury, but not the underlying cause of it. Further research is needed on guidelines for diagnosing type 1 and type 2 MI as well as management of MI in the setting of a recent hemorrhage.
